# Description of Immature Stages and Life Cycle of the Treehopper, *Guayaquila projecta*

**DOI:** 10.1673/031.010.19901

**Published:** 2010-11-17

**Authors:** Mario Alfredo Linares, Lilia Estela Neder, Chris Dietrich

**Affiliations:** ^1^ Instituto de Biologia de la Altura, Universidad Nacional de Jujuy. Av. Bolivia 1661 (4600) San Salvador de Jujuy, Jujuy, Argentina; ^2^ Instituto de Biologíía de la Altura, Universidad Nacional de Jujuy, CONICET. Argentina; ^3^ Illinois Natural History Survey, 1816 S. Oak St., Urbana, IL 61820, USA

**Keywords:** Aconophorini, *Bougainvillea glabra*, Hemiptera, Homoptera, Membracidae, morphology, ontogeny

## Abstract

Immature stages of the membracid *Guayaquila projecta* (Funkhouser) (Hemiptera: Cicadomorpha: Membracidae), collected in San Salvador de Jujuy, Argentina on *Bougainvillea glabra* Choisy (Caryophyllales: Nyctaginaceae), are described in detail based on specimens reared in the laboratory. Like other membracids, this species has five nymphal instars, not seven as previously reported. Morphological characters for identifying the different instars of *G. projecta*, determining the sex of later instars and distinguishing this species from other members of the *Guayaquila pugnax* group, are discussed. At 19 ±± 4°°C, RH 59 ±± 9%, and a 12:12 L:D photoperiod, the time required for development from egg to adult emergence was 73 ±± 5 days.

## Resumen

En San Salvador de Jujuy (Jujuy, Argentina) se detectóó la presencia de ejemplares de *Guayaquila projecta* (Funkhouser) (Hemiptera: Cicadomorpha: Membracidae) sobre la planta ornamental *Bougainvillea glabra* Choisy (Caryophyllales: Nyctaginaceae). Se describen detalladamente los estados inmaduros basados en ejemplares criados en laboratorio. Se confirma que esta especie atraviesa por cinco estadios ninfales al igual que otros membráácidos, contrastando con un trabajo previo que indica la presencia de siete. Se discute la utilizacióón de determinados caracteres morfolóógicos para diferenciar los estadios, distinguir los sexos en los úúltimos estadios y separar esta especie de los otros miembros del grupo *Guayaquila pugnax.* A 19 ±± 4°°C, RH 59 ±± 9% y fotoperiodo 12hs luz-12hs oscuridad, el tiempo requerido para el desarrollo de huevo hasta la emergencia del adulto fue de 73 ±± 5 díías.

## Introduction

The family Membracidae Rafinesque (Hemiptera: Cicadomorpha) includes about 3100 species classified into 9 subfamilies and 48 tribes worldwide (McKamey 1998, Cryan *et al.* 2000, Wallace and Deitz 2004). It is the most diverse in the Neotropical region. Dietrich and Deitz (1991) revised the tribe Aconophorini (Membracinae), species of which are distributed throughout the Neotropics. According to this revision the tribe has 51 species grouped into 3 genera: *Guayaquila* Goding, *Calloconophora* Dietrich, and *Aconophora* Fairmaire. The genus *Guayaquila* has 22 species that are included in 12 groups. One of them, the Pugnax group, includes *G. pugnax* (Germar), *G. peruviensis* (Dietrich), and *G. projecta* (Funkhouser). The last is the only species recorded from Argentina, in the provinces of Catamarca, Salta, and Tucumáán.

In San Salvador de Jujuy, Jujuy, Argentina, (Longitude: 65°° 18′? W, Latitude: 24°° 11′? S, 1250 masl) *G. projecta* has been found on *Bougainvillea glabra* Choisy (Caryophyllales: Nyctaginaceae), commonly known as ““Santa Rita””, an ornamental plant which is widely distributed in parks and gardens in San Salvador de Jujuy.

Previously Marcus (1950) reared nymphs of *G. projecta* from Bolivia and reported seven nymphal instars, a departure from the usual five instars in other Membracidae. The aim of this work is to investigate the life cycle of *G. projecta* in more detail and to analyze the morphological characteristics useful for differentiating its different stages and instars.

## Materials and Methods

### Life cycle

Egg-layings were obtained under laboratory conditions. Ten newborn nymphs were put on each of ten freshly cut branches of *B. glabra* and then placed in breeding chambers at 19 ±± 4°°C, RH 59 ±± 9%, and 12:12 L:D photoperiod. The branches were periodically renewed. The duration of different nymphal instars and adult longevity were recorded.

### Morphology

The morphological characteristics of eggs dissected from the ovaries of gravid females, eggs recently laid, and mature eggs were studied. Morphological characteristics of the first-instar nymph were analyzed in detail, and only the observed changes were added to the descriptions of later instars. Color was described based on examination of living specimens anesthetized with sulfuric ether. Preparations were measured microscopically using a stereo-microscope. Reported lengths were measured on the midline and reported width is the maximum for a given structure. Chaetotaxy is reported for the left half of the body.

Reported measurements are averages for 10 individuals. All the dimensions are given in millimeters. A camera lucida on a Reichert microscope was used to make the drawings.

To corroborate the number of nymphal instars linear regression analyses were carried out for nine measurements: head length and width, rostrum length, interocular distance, ocular diameter, meso and metathorax length, and tibia III maximum width and length. Also, the relationship between the rostrum length and the total length of the body and the growth factor for those characters was calculated as the difference between the average value of an instar and that of the previous one.

The *G. projecta* studied specimens were kept as part of the collection at the Instituto de Biologíía de la Altura, and the *B. glabra* material was kept at the herbarium of the School of Agrarian Sciences- UNJu (National University of Jujuy) under number 2469 (JUA).

## Results

### Life cycle duration

Individuals required an average of 75 days to go from egg to adult, with each instar requiring 7–10 days on average (Table 1). The longevity of the adults varied to a maximum of 60 days.

**Figure 1.**
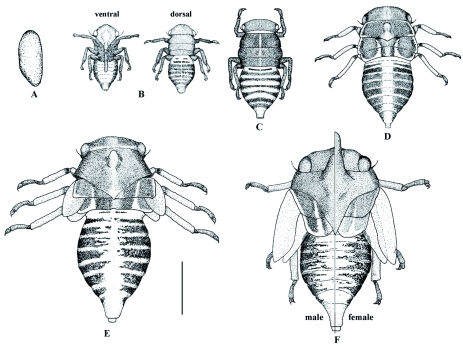
Immature stages of *G.* *projecta* (A) egg; (B) first instar nymph; (C) second instar nymph; (D) third instar nymph; (E) fourth instar nymph; (F) fifth instar nymph showing characteristics of males and females. Scale = 1 mm. High quality figures are available online.

Description of the immature stages (Figure 1)

**Egg.** The mature ovarian eggs were 0.95 ±± 0.03 mm long and 0.35 ±± 0.03 mm wide. When newly deposited they were white and 0.99 ±± 0.05 mm long and 0.38 ±± 0.02 mm wide. They were subcylindrical with the basal end sharp and the distal end rounded (Figure 1.1). The chorion was translucent and smooth. The average egg mass length was 8 mm and its width was 3.5 mm. The average number of eggs laid was 45, with a range of 24 to 72.

**First instar nymph.** Elongate, subovoid (Figure 1 B). Color when newly emerged from egg (or recently molted later instars) yellow with transparent legs, and ventral part of abdomen reddish orange. When fully sclerotized, head, thorax, legs and dorsal pleural part of abdomen blackish brown. Abdominal sterna reddish brown. Thin yellowish white middorsal line extended from vertex to 7th abdominal segment.

**Table 1.**
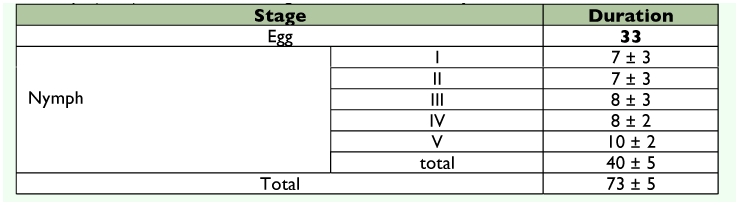
Duration in days (±± SE) of the different stages and instars of *G.* *Projecta.*

**Figure 2.**
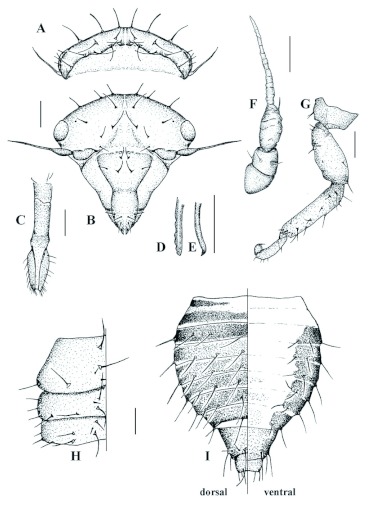
First instar nymph of *G.* *projecta* (A) head, dorsal view; (B) head, ventral view; (C) head, rostrum; (D) head, mandibular stylet; (E) head, maxillary stylet; (F) head, antenna; (G) thorax, dorsal view; (H) third leg; (I) abdomen, dorsal view and ventral view. Scale = 0.05 mm. High quality figures are available online.

Head triangular with eyes red. (Figure 2 B). Upper margin of vertex visible dorsally. Vertex wide with medial area slightly depressed. Five setae on posterior edge, two setae in middle, and three bigger setae along anterior margin, in dorsal view (Figure 2 A). Epicranial groove bifurcated and extended to antennal insertion in ventral view (Figure 2 B). Two medial setae, two between groove and eyes and one near antennal base. Clypeus convex. Postclypeus trapezoidal with a flat superior margin; 1.3 times wider than long; with two setae in midline and one on lateral edge. Anteclypeus subovoid, 1.5 times longer than wide with two divergent setae at top of inferior third and subterminal one. Rostrum extended from coxae between first and second pair of legs to sixth abdominal segment (Figure 2 C). Terminal segment of rostrum 2.3 times longer than wide with many lateral setae. Mandibular stylet (Figure 2 D) with five blunt distal teeth. Maxillary stylets longer than anterior ones, with tips slightly curved (Figure 2 E). Antennae setiform (Figure 2 F), inserted at junction between clypeus, vertex and gena and extended laterally. Inverted cone shaped first antennule without evident sensoria; cylindrical second antennule narrower than length (0.98:1) and, at midlength with four setae placed around it. Third antennule peduncular, with widened basal portion and distal flagellum; external surface with group of sensory elements formed by two setae, a middle one and a sensory cone next to base of flagellum and 5 ring shaped rows of sensory receptors. Flagellum tapered with apparent segmentation formed by various ring-shaped sensory receptors; 1.7 times length of the first and second antennules together.

Thorax (Figure 2 G): Slightly trapezoidal pronotum with largest base in contact with mesonotum, length 0.47 times total length of tagma. Each side of pronotum with 6 setae: 1 near anterior edge along midline, 3 near posterior edge along midline, one near midpoint along posterior margin, and one lateral near posterior margin. Mesonotum rectangular, 0.56 times length of the pronotum with setae distributed similarly to those of prothorax but with three additional lateral setae (anterior, medial and posterior). Metanotum different from the previous segment in lacking anterior lateral seta and having one more sub-medial seta. Legs (Figure 2 H): coxae transversally placed with a slightly convex internal surface, first pair longer than wide in contrast to other pairs. Femora widened. Tibiae flattened. Tarsi two segmented. Chaetotaxy of all legs similar: tibia with five setae on the anterior margin, 2 on the posterior margin, 4 sub-medial ones and 4 setae on distal margin; tarsi with first segment bearing two posterior and one medial setae, second segment with three setae anterior, 3 posterior and 2 sub-medial setae; pretarsus with seta at base of each claw.

Abdomen pyriform with maximum width between third and fourth segment (Figure 2 I). First two segments weakly sclerotised. Segments 3 to 8 with similar chaetotaxy: 3 dorsal, 1 lateral and 2 ventro-lateral setae (Figure 2 I dorsal-ventral). Length of segments subequal except 9^th^ which is 1.7 times longer than previous segment and with three pairs of setae, dorsal, lateral and ventral, each pair with one long and one short seta. 10th uromere cylindrical; length 1.4 times width, retracted within 9^th^ segment (telescopic), with eight-seta posterior ring. Spiracles present on 3^rd^ to 8^th^ abdominal segments.

**Second instar Nymph (Figure 1 C).** Color pattern of the first instar nymph maintained, with ventral portion of abdomen reddish orange on sides and middle part yellow.

General proportions of body maintained. Rostrum extended to posterior extreme of fourth abdominal segment. Coxae with 1 to 3 long setae.

Chaetotaxy as in first instar.

**Third instar nymph (Figure 1 D).** Thorax lateral margins with white wax band that extends along metathorax posterior margin to sublateral region and dorsally towards head, reaching prothorax posterior edge. Adjacent area to midline lighter forming yellowish band. Legs with body of femur, tibia proximal extreme, and tarsi dark brown. Distal part of femur and tibiae yellowish.

Rostrum not extended beyond third abdominal segment.

Pronotum with anterior horn present as button shaped enlargement at anterior third. Posterior margin sharpened into posterior process, extended to middle of mesonotum. Mesonotum with lateral extensions (wing pads).

Coxae with strong setae on external lateral surface.

General pilosity better developed, obscuring original chaetotaxy

**Table 2.**
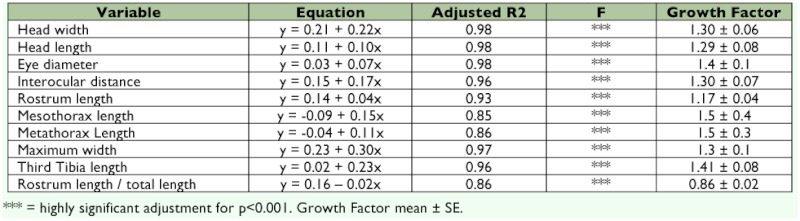
Linear regression of the morphological characteristic and morphometric relationship in nymphs of *G.* *projecta.*

**Fourth instar nymph (Figure 1 E).** Wing pad apices waxen white. Rest of body color similar to that of third instar.

Ocelli visible.

Body with general pilosity abundant on prontum and regularly distributed. Horn, in dorsal view, reaching pronotum anterior edge. Posterior process covering entire mesonotum. Wing pads extended to second abdominal segment.

Genital plates evident between the eighth and ninth abdominal segments.

**Fifth instar nymph (Figure 1 F).** Horn brownish. Posterior pronotal process with reddish extreme extended to first abdominal segment.

Horn length sexually dimorphic, shorter in males and not exceeding head anterior edge. Wing pads extended to fifth abdominal segment. Wing veins evident. The tibial spines of third pair of legs evident. Genital plates larger but more complex structures not evident

### Morphometric relationships and growth factor.

Linear regressions show a highly significant adjustment for a linear function in all analyzed measurements. The adjusted R^2^ values are shown in Table 2 in which an average growth factor is also evident. These values were highly stable throughout the nymphal development of *G. projecta.*

## Discussion

Morphological characters distinguishing the first two instars of *G. projecta* are: the dorsal color, the general length of the body, the placement of the apical rostrum segment in relation to the body, and the chaetotaxy. Beginning with the third stage, the relative development of the wing pads and pronotum become more important. The length of the pronotum increases progressively because of the formation of the horn and the posterior process that, in the adults, may cover the whole abdomen.

In the fifth instar nymph, the sexes may be distinguished by the horn length.

The brood obtained under controlled laboratory conditions has clarified details of the life cycle: 73 ±± 5 days (egg-adult) duration, 44% of which corresponds to the egg state and, as in other membracids, five nymphal instars. The results of this study contrast with those of Marcus (1950), who reported seven instars for *G. projecta* (=*Aconophora projecta*, Funkhouser) based on differences in size.

The use of linear regression analysis helped to corroborate the five instars. Other studies of the immature stages of Membracoidea have used similar analyses. To identify the nymphal instars of *Sextius virescens* (Fairmaire) (Cicadellidae), Kitching (1974) used the proportion of the head width to the total width of the nymph; Page (1979) used the growth factor of *Austroasca viridigrisea* (Paoli) (Cicadellidae) to characterize this species in relation to the size increase of the cephalic capsule. Both kinds of analysis were carried out for *G. projecta* and the growth factor was very similar between instars in many of the analyzed characteristics such as rostrum length/total length, rostrum length, and head width. Thus, as also seems to be true for many other Hemiptera (Page 1979; Segnini and Montagne 1986; Nixon and Thompson 1987; Klingenberg and Zimmerman 1992; Viscarret and Botto, 1997; Rodrigues et al 2005; Setamou and Jones 2005; Rodriguez and Peck 2007), nymphal growth in *G. projecta* appears to obey Dyar's rule which was originally proposed for Lepidoptera (Page 1979).

*Guayaquila projecta* is found in Bolivia in Cochabamba (Marcus 1950), La Paz, Sorata; in Brazil in Paranáá; in Colombia in La Cumboc; in Paraguay in San Pedro; and in Argentina in Tucumáán, Salta, and Catamarca (Dietrich and Deitz 1991). In this study the distribution is extended to the province of Jujuy (Argentina).

Dietrich and Deitz (1991) record *Mimosa lorentzii* (Fabaceae) as a host plant and this study adds a new host record: *Bougainvillea glabra.*
